# Nrf2 Activation Alleviates Silica-Induced Toxicity in Alveolar Macrophages via Glutamine Metabolic Reprogramming

**DOI:** 10.3390/toxics14070602

**Published:** 2026-07-10

**Authors:** Xinyi Zhu, Jixia Hu, Fangguo Lu, Ziyi Liu, Zhibin Wang, Chang Liu, Quan Zhu, Jun Lu

**Affiliations:** 1 Department of Preventive Medicine, School of Medicine, Hunan University of Chinese Medicine, Changsha 410208, China; 202308020345@stu.hnucm.edu.cn (X.Z.);; 2 Department of Pathogenic Biology, School of Medicine, Hunan University of Chinese Medicine, Changsha 410208, China; 3 Department of Basic Medicine, School of Integrated Traditional Chinese and Western Medicine, Hunan University of Chinese Medicine, Changsha 410208, China; 4 Department of Immunology, School of Medicine, Hunan University of Chinese Medicine, Changsha 410208, China

**Keywords:** crystalline silica, alveolar macrophages, Nrf2, metabolic reprogramming, M1 phenotype

## Abstract

As a major occupational hazard, crystalline silica (SiO_2_) poses a severe risk of pulmonary toxicity. While the irreversible fibrosis of late-stage silicosis has been extensively studied, the cellular and molecular mechanisms by which SiO_2_ reprograms macrophage metabolism to drive early pathogenesis remain poorly understood. To elucidate this early immune-inflammatory response, we combined targeted metabolomics, pharmacological treatments, and nutrient deprivation in murine alveolar macrophages. Our results demonstrate that SiO_2_ exposure severely impairs the master antioxidant regulator, nuclear factor erythroid 2-related factor 2 (Nrf2), triggering excessive reactive oxygen species (ROS) accumulation and upregulated glutamine catabolism to drive pro-inflammatory M1 macrophage polarization. We demonstrated that Nrf2 activation with tert-butylhydroquinone (TBHQ) redirected glutamine metabolic flux from pro-inflammatory catabolism to antioxidant anabolism, significantly attenuating SiO_2_-induced M1 polarization. Conversely, Nrf2 inhibition via ML385 exacerbated the inflammatory response. Furthermore, introducing a glutamine deprivation (−Gln) model revealed that restricting glutamine availability significantly attenuated the ability of Nrf2 to reverse M1 polarization, suggesting that its immune-protective effects largely depend on an intact glutamine metabolic pathway. Ultimately, our findings underscore the severe risks of silica exposure and identify the Nrf2–glutamine metabolic axis as a promising target, providing novel mechanistic insights and a robust basis for “antioxidant–metabolic” dual-target interventions in early-stage silicosis.

## 1. Introduction

The health risks associated with dust exposure remain a severe global concern. Various forms of pneumoconiosis account for more than 18,000 deaths annually, with silicosis imposing a particularly profound burden, as the global caseload now exceeds 2.64 million [1,2]. Once silica particles enter the lungs via the respiratory tract, alveolar macrophages (AMs)—acting as the body’s first line of innate immune defense against inhaled dust—initiate clearance mechanisms via phagocytosis [3]. However, upon the internalization of excessive silica particles, subsequent lysosomal rupture precipitates a vicious cycle of “phagocytosis–damage–rephagocytosis,” thereby transforming these macrophages from a defensive barrier into a pathological hub that drives oxidative stress and persistent inflammation [4,5]. Given the critical reversible window prior to the establishment of fibrosis in silicosis, reprogramming the phenotype of AMs during this phase to block the inflammatory cascade is pivotal for achieving early reversal [6]. Therefore, elucidating the core regulatory mechanisms of AMs in SiO_2_-induced early-stage injury holds profound scientific and clinical value for identifying intervention targets in the pre-fibrotic stage and halting the pathological progression of silicosis.

Accumulating evidence demonstrates that an imbalance in AM phenotypic polarization is a central mechanism underlying the pathological progression of silicosis, with the hyperactivation of the pro-inflammatory M1 phenotype acting as the primary driver of early lung parenchymal damage [7]. Upon initial SiO_2_ exposure, an acute oxidative burst acts as a critical signaling event that drives the rapid polarization of AMs toward the M1 phenotype, marked by robust expression of CD86 and associated markers [6,8]. This unchecked M1 activation provokes the continuous secretion of tumor necrosis factor-α (TNF-α), interleukin-6 (IL-6), and reactive oxygen species (ROS), culminating in a severe inflammatory storm that directly injures alveolar epithelial cells [4]. Without early intervention to reverse this M1-mediated activation, the unbridled inflammatory cascade persists, propagating a vicious cycle of sustained alveolar damage and disease progression [7,9]. Consequently, identifying regulatory factors that can effectively restore redox homeostasis and reprogram macrophage polarization represents a paramount strategy for arresting the early pathogenesis of silicosis.

As a master transcription factor, nuclear factor E2-related factor 2 (Nrf2) orchestrates cellular redox balance, metabolic flux, and detoxification defenses via the Nrf2-Keap1-ARE pathway [10,11]. Under baseline physiological conditions, Nrf2 is sequestered in the cytoplasm by Kelch-like ECH-associated protein 1 (Keap1). Upon encountering stress, however, Nrf2 binds to and activates the antioxidant response element (ARE), driving the transcription of downstream target genes such as heme oxygenase-1 (HO-1) and glutathione (GSH) synthase, thereby constructing a formidable antioxidant defense network [12,13]. Beyond its canonical role in directly neutralizing oxidative damage, the Nrf2 signaling pathway exerts profound immunomodulatory effects by suppressing macrophage M1 polarization and facilitating their transition toward an anti-inflammatory phenotype [14]. Strikingly, SiO_2_ exposure fundamentally abrogates the initiation of this endogenous antioxidant program by impeding Nrf2 nuclear translocation and triggering its proteolytic degradation. The ensuing collapse of this central defense hub permits persistent ROS accumulation, which subsequently ignites an inflammatory cascade and exacerbates pulmonary injury [15,16]. Consequently, therapeutically reactivating the Nrf2 pathway emerges as a highly promising strategy for ameliorating early-stage pathogenesis in silicosis.

Furthermore, Nrf2 has emerged as a master regulator of immunometabolic reprogramming [11]. Our previous in vivo studies have demonstrated that SiO_2_ exposure not only triggers severe oxidative stress in rat lungs but also profoundly disrupts pulmonary metabolic homeostasis [17,18]. Notably, this disruption features the dysregulation of metabolites such as glutamine, a perturbation highly correlated with the progressive impairment of Nrf2 signaling [19]. Because alveolar macrophages serve as the primary effector cells against dust toxicity, their immunometabolic reprogramming plays a critical role in silica-induced pathogenesis. Macrophages generally rely on the SLC1A5 transporter to internalize extracellular glutamine, which is subsequently converted into glutamate by the rate-limiting enzyme GLS1 to fuel de novo GSH synthesis and sustain redox balance [20]. However, under severe stress conditions, pathological hyperactivation of this SLC1A5/GLS1-driven catabolic pathway actively propels macrophages toward a pro-inflammatory M1 phenotype [21]. There is a recognized, tight bidirectional crosstalk between Nrf2 and the glutamine metabolic network: Nrf2 deficiency causes profound dysregulation in macrophage glutamine utilization, while an aberrant glutamine metabolic state can reciprocally undermine Nrf2 protein stability via post-translational modifications [22,23,24]. Despite this, it remains unclear whether Nrf2 can target this specific axis to remodel immunometabolism following SiO_2_ exposure. We therefore hypothesized that the targeted activation of Nrf2 could redirect macrophage glutamine metabolic flux—diverting it from pro-inflammatory catabolism back toward antioxidant anabolism—thereby abrogating pathological M1 polarization. Consequently, this study focuses on elucidating the immunometabolic regulatory role of Nrf2 in mitigating SiO_2_-induced alveolar macrophage injury. By deciphering the molecular mechanisms underlying Nrf2-mediated reprogramming of the glutamine pathway, this work aims to provide a robust, forward-looking framework for the future clinical exploration of “antioxidant–metabolic dual-target” therapeutic strategies in early-stage silicosis.

## 2. Materials and Methods

### 2.1. Preparation and Characterization of SiO_2_ Particles

Silicon dioxide (SiO_2_) particles were purchased from Sigma-Aldrich (St. Louis, MO, USA; CAS No. 14808-60-7). In line with the manufacturer’s specifications and our previously reported data [17], silica is a white-to-off-white powder with a particle size distribution ranging from 0.5 to 10 µm; notably, approximately 80% of the particles fall within the respirable 1–5 µm range. Our prior scanning electron microscopy (SEM) analyses confirmed that these particles predominantly feature near-spherical and polyhedral geometries, alongside a natural tendency to aggregate [17]. To prepare the experimental stock suspension, SiO_2_ was dispersed in distilled water and autoclaved at 120 °C for 20 min, a step that also effectively prevented microbe-mediated clumping.

### 2.2. Cell Culture

The murine alveolar macrophage cell line (MH-S) and all associated cell culture reagents, including RPMI-1640 medium (Cat. No. PM150110), fetal bovine serum (FBS; Cat. No. 164210-50), and a penicillin–streptomycin mixture (Cat. No. PB180120), were obtained from Procell Life Science & Technology Co., Ltd. (Wuhan, China). The cells were cultured in RPMI-1640 medium supplemented with 10% FBS and 1% penicillin–streptomycin. All cultures were maintained in a standard humidified incubator at 37 °C under an atmosphere containing 5% CO_2_.

### 2.3. Cell Viability Assay

Log-phase MH-S cells were seeded into 96-well plates at a density of 1 × 10^4^ cells/well (100 µL/well). Following overnight adherence, the cells were exposed to either the vehicle control (saline) or 100 µg/mL of SiO_2_ for 24 h. After the designated treatment period, the original medium was discarded, and each well was replenished with 100 µL of fresh culture medium containing 10% Cell Counting Kit-8 (CCK-8) reagent (Cat. No. C6005; NCM Biotech, Suzhou, China). The plates were then incubated in the dark at 37 °C under 5% CO_2_ for an additional 2 h. Finally, the absorbance at 450 nm was recorded using a microplate reader.

### 2.4. Measurement of Intracellular ROS Levels

Log-phase MH-S cells were seeded into 24-well plates at a density of 3 × 10^5^ cells/mL. Following adherence and the respective pharmacological treatments, the cells were washed three times with PBS. The cells were then incubated with a 10 µM DCFH-DA fluorescent probe (Cat. No. E004-1-1; Nanjing Jiancheng Bioengineering Institute, Nanjing, China) in the dark at 37 °C for 30 min. After washing away the unbound probe, intracellular green fluorescence was directly quantified using a microplate reader at an excitation wavelength of 488 nm and an emission wavelength of 525 nm.

### 2.5. Determination of Cellular Malondialdehyde (MDA) Levels

Lipid peroxidation was evaluated using an MDA assay kit (Cat. No. A003-1; Nanjing Jiancheng Bioengineering Institute). Harvested cells were sonicated on ice, and the resulting homogenates were mixed with the assay working solution prior to a 40 min incubation in a 95 °C water bath. Upon cooling, the mixtures were centrifuged at 4000 rpm for 10 min. The supernatant was then transferred to a 96-well plate, and the absorbance was measured at 532 nm. Total protein concentrations were simultaneously quantified via the BCA assay for data normalization, with final MDA contents expressed as nmol/mg protein.

### 2.6. Measurement of the Intracellular GSH/GSSG Ratio

Intracellular total glutathione (T-GSH) and oxidized glutathione (GSSG) levels were quantified using a glutathione assay kit (Cat. No. A005; Nanjing Jiancheng Bioengineering Institute). Cells from each experimental group were lysed with the provided extraction buffer via sonication on ice, followed by centrifugation at 3500 rpm for 10 min at 4 °C. The absorbance of T-GSH and GSSG in the supernatants was measured at 405 nm according to the manufacturer’s protocols. Protein concentrations were determined using the BCA assay for normalization. The reduced GSH content was calculated using the following formula: GSH = T-GSH − 2 × GSSG.

### 2.7. Enzyme-Linked Immunosorbent Assay (ELISA)

MH-S cells in the exponential growth phase were seeded into culture plates at an appropriate density. Following complete adherence and respective pharmacological interventions, the culture supernatants were collected and centrifuged at 3000 rpm for 10 min to remove cellular debris. The concentrations of TNF-α and IL-1β in the supernatants were determined using specific ELISA kits (TNF-α: Cat. No. YJ002095A; IL-1β: Cat. No. EK201B; both from Shanghai Enzyme-linked Biotechnology Co., Ltd., Shanghai, China) in strict accordance with the manufacturer’s instructions.

### 2.8. Immunofluorescence Staining

MH-S cells were seeded onto coverslips in 12-well plates at a density of 3.5 × 10^5^ cells/well and incubated for 12 h to allow for complete adherence before pharmacological interventions. Following the treatments, the cells were fixed with 4% paraformaldehyde (PFA) for 30 min at room temperature, permeabilized with Triton X-100 for 10 min, and blocked with 5% goat serum. Subsequently, the coverslips were incubated overnight at 4 °C with the following specific rabbit primary antibodies: anti-Nrf2 (1:100; Cat. No. HA721432), anti-CD206 (1:100; Cat. No. ET1702-04), and anti-CD86 (1:500; Cat. No. ET1606-50) (all from HuaBio, Hangzhou, China), as well as anti-GLS1 (1:500; Cat. No. 81486-1-RR) and anti-SLC1A5 (1:500; Cat. No. 20350-1-AP) (both from Proteintech, Wuhan, China). The following day, after washing with PBS, the cells were incubated with an Alexa Fluor 488-conjugated goat anti-rabbit secondary antibody in the dark at room temperature for 90 min. The nuclei were counterstained with DAPI (1 µg/mL) for 10 min, and the coverslips were subsequently mounted using an antifade mounting medium. Finally, the slides were visualized and imaged utilizing an Olympus confocal microscope at excitation wavelengths of 488 nm and 405 nm.

### 2.9. Reverse Transcription Quantitative Polymerase Chain Reaction (RT-qPCR)

Total RNA was extracted from the MH-S cells in each group using the SteadyPure RNA Extraction Kit (Cat. No. AG21023; Accurate Biology, Changsha, China). Following spectrophotometric determination of RNA concentration and purity, 2000 ng of total RNA was reverse-transcribed into single-stranded cDNA utilizing the NovoScript Plus Reverse Transcription Kit (Cat. No. E047-01B; Novoprotein, Suzhou, China). Subsequently, amplification and melting curve analyses were performed on a real-time PCR system using the 2× NovoStart SYBR qPCR SuperMix (Cat. No. E096-01A; Novoprotein, Suzhou, China). All samples were analyzed in technical triplicate, with β-actin and GAPDH serving as endogenous reference genes. The relative expression levels of the target genes were calculated using the $2^{-\Delta \Delta C_{T}}$ method. The primer sequences utilized for each target gene are detailed in Table 1.

### 2.10. Determination of Glutamate (Glu) Content

The cell pellets from each group were collected and lysed in the provided extraction buffer at a concentration of 5 × 10^6^ cells/mL via ultrasonic homogenization on ice. Following the addition of a protein precipitant, the lysates were centrifuged at 12,000 rpm for 10 min at 4 °C. The glutamate (Glu) levels in the resulting supernatants were quantified utilizing a glutamate assay kit (Cat. No. BC1580; Solarbio, Beijing, China) in strict accordance with the manufacturer’s instructions. Finally, the absorbance was measured at a wavelength of 450 nm using a spectrophotometer, and the final concentrations were calculated based on a standard curve.

### 2.11. Targeted Metabolomics Analysis

MH-S cells were randomly assigned to three experimental groups (*n* = 5 biological replicates per group): a control group, a model group (exposed to 100 µg/mL SiO_2_), and a TBHQ intervention group (co-treated with SiO_2_ and TBHQ for 24 h). Following collection, cell pellets (approximately 1 × 10^7^ cells per sample) were snap-frozen in liquid nitrogen and subsequently homogenized in 500 µL of a pre-chilled methanol/water extraction buffer (4:1, *v*/*v*). To ensure comprehensive cellular disruption and metabolite extraction, the samples were subjected to three successive freeze–thaw cycles in liquid nitrogen, followed by ultrasonication on ice for 15 min. The homogenates were then incubated at −40 °C for 1 h to facilitate complete protein precipitation. Following high-speed centrifugation (12,000 rpm for 15 min at 4 °C), the resulting supernatants were carefully collected for immediate instrumental analysis. Targeted metabolomic profiling was executed utilizing an Agilent 1290 UHPLC system (Agilent Technologies, Santa Clara, CA, USA) seamlessly coupled to an AB Sciex 6500+ tandem mass spectrometer (AB Sciex, Framingham, MA, USA). Depending on the specific physicochemical properties of the target analytes, chromatographic separation was achieved using either an ACQUITY BEH C18 reversed-phase column or an Atlantis Z-HILIC column (both from Waters Corporation, Milford, MA, USA). The injection volume was standardized at 2 µL. Mass spectrometric data acquisition was conducted in multiple reaction monitoring (MRM) mode, employing an electrospray ionization (ESI) source. Raw data acquisition and absolute quantification were meticulously processed using SCIEX Analyst (AB Sciex) and BioBud (version 2.0.4) software platforms. To evaluate macroscopic shifts in metabolic profiles across the experimental groups, the normalized datasets were subjected to principal component analysis (PCA) and Partial Least Squares-Discriminant Analysis (PLS-DA). Furthermore, differential metabolic pathways were comprehensively parsed utilizing a combination of volcano plots, hierarchical clustering heatmaps, and KEGG enrichment analyses. Finally, Spearman’s correlation analysis was deployed to map the intricate biological interactions between core differential metabolites and key phenotypic markers (i.e., ROS, CD86, and CD206).

### 2.12. Western Blot

Following collection, MH-S cells were lysed on ice for 30 min using RIPA lysis buffer (Cat. No. ES-8148; ECOTOP, Guangzhou, China) supplemented with a 1% protease inhibitor cocktail (Cat. No. SW106-02; Sevenbio, Beijing, China). The lysates were then centrifuged at 12,000 rpm for 10 min at 4 °C, and the resulting supernatants were carefully collected. Total protein concentrations were determined utilizing a Micro BCA Protein Assay Kit (Cat. No. SW201-02; Sevenbio, Beijing, China). Equal amounts of denatured protein (30 µg per lane) were resolved by SDS-PAGE and subsequently transferred onto PVDF membranes under constant current. After blocking with 5% non-fat milk for 1.5 h, the membranes were incubated overnight (12–14 h) at 4 °C with the following specific rabbit primary antibodies: anti-Nrf2 (1:7000; Cat. No. 16396-1-AP), anti-GLS1 (1:25,000; Cat. No. 81486-1-RR), and anti-SLC1A5 (1:10,000; Cat. No. 20350-1-AP) (all from Proteintech, Wuhan, China); anti-HO-1 (1:2000; Cat. No. AF5393), anti-Keap1 (1:2000; Cat. No. AF5266) (all from Affinity Biosciences, Cincinnati, OH, USA); anti-CD206 (1:2500; Cat. No. ET1702-04) and anti-CD86 (1:10,000; Cat. No. ET1606-50) (both from HuaBio, Hangzhou, China); and anti-β-actin (1:100,000; Cat. No. AC026; ABclonal, Wuhan, China). The following day, after washing three times with TBST, the membranes were incubated with an HRP-conjugated secondary antibody for 1.5 h at room temperature. Finally, the protein bands were visualized using an enhanced chemiluminescence (ECL) substrate, and the relative densitometric values of the target bands were quantified utilizing ImageJ software (version 1.48v).

### 2.13. Statistical Analysis

Statistical evaluations and graphical representations of the experimental data were performed utilizing SPSS software (version 25.0; IBM Corp., Armonk, NY, USA) and GraphPad Prism (version 9.5; GraphPad Software, San Diego, CA, USA), respectively. Data are expressed as mean ± standard deviation (SD). Two-group comparisons were conducted using unpaired two-tailed Student’s *t*-tests, Welch’s *t*-tests (for unequal variances), or Mann–Whitney U tests (for non-normally distributed data). For multiple group comparisons, normally distributed data with equal variances were analyzed using one-way ANOVA with an LSD post hoc test. Data violating variance homogeneity or normality were evaluated via Welch’s ANOVA or the Kruskal–Wallis test (with Dunn’s post hoc analysis), respectively. Statistical significance between groups is denoted as follows: “ns” indicates no significant difference; * *p* < 0.05; ** *p* < 0.01; *** *p* < 0.001; and **** *p* < 0.0001. All experiments were performed in at least three independent biological replicates.

## 3. Results

### 3.1. SiO_2_ Suppresses Nrf2 Expression and Induces Oxidative Stress and M1 Polarization in Murine Alveolar Macrophages

**Figure 1:**
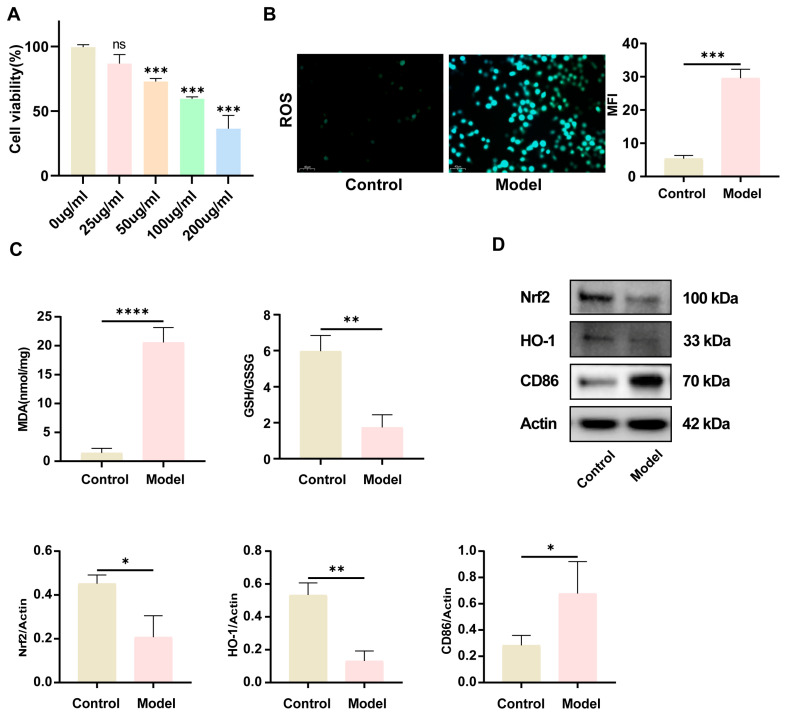
SiO_2_ induces redox imbalance and M1 polarization in MH-S alveolar macrophages: (**A**) Cell viability was assessed via an MTT assay. Exposure of cells to varying concentrations of SiO_2_ (0, 25, 50, 100, and 200 µg/mL) for 24 h resulted in a dose-dependent decrease in the cell survival rate. (**B**) Representative fluorescence microscopy images depicting intracellular ROS accumulation in the Control and Model (100 µg/mL SiO_2_) groups, accompanied by the quantitative analysis of mean fluorescence intensity (MFI). (**C**) Determination of MDA content (left panel) and the GSH/GSSG ratio imbalance (right panel) in MH-S cells using biochemical assay kits. Data in the bar graphs (**C**) are derived from six independent experiments (*n* = 6). (**D**) Representative Western blot images and corresponding densitometric analyses illustrating the effects of 100 µg/mL SiO_2_ exposure on the protein expression levels of Nrf-2, HO-1, and the M1 marker CD86. Data in bar graphs (**B**,**D**) are presented as the mean ± standard deviation (SD) of three independent experiments. Statistical significance for all groups was evaluated relative to the control group. Ns, not significant; * *p* < 0.05, ** *p* < 0.01, *** *p* < 0.001, and **** *p* < 0.0001.

Murine alveolar macrophages (MH-S) were exposed to a range of SiO_2_ concentrations (25, 50, 100, and 200 µg/mL) to identify the optimal dose for establishing the in vitro injury model. Cell viability exhibited a significant dose-dependent decline compared to the control group (Figure 1A). During the dose-screening phase, 50 µg/mL induced only mild cytotoxicity, which proved insufficient to fully trigger the targeted pathological cascades. Conversely, exposure to 200 µg/mL precipitated profound cell death, leaving an inadequate viable cell population for subsequent metabolic and phenotypic evaluations. Notably, treatment with 100 µg/mL reliably reduced cell viability to approximately 60%, successfully inflicting robust cellular damage while preserving sufficient live cells for downstream mechanistic investigations. Consequently, 100 µg/mL was adopted as the standard working concentration for this study. To evaluate the capacity of SiO_2_ to disrupt cellular redox homeostasis, key intracellular oxidative stress indicators were systematically quantified. Compared with the control group, SiO_2_ treatment provoked a pronounced elevation in both MDA levels and ROS fluorescence intensity, accompanied by a precipitous decline in the intracellular GSH/GSSG ratio (Figure 1B,C). Furthermore, SiO_2_ exposure significantly downregulated the protein expression of Nrf2 and HO-1, while concomitantly upregulating the M1 macrophage marker CD86 (Figure 1D). Taken together, these findings demonstrate that SiO_2_ inflicts severe oxidative injury and drives M1 polarization in MH-S cells, fundamentally by impairing Nrf2-mediated antioxidant defenses.

### 3.2. Nrf2 Activation Reverses Silica-Induced Oxidative Stress and Reprograms M1 Polarization in Alveolar Macrophages

**Figure 2:**
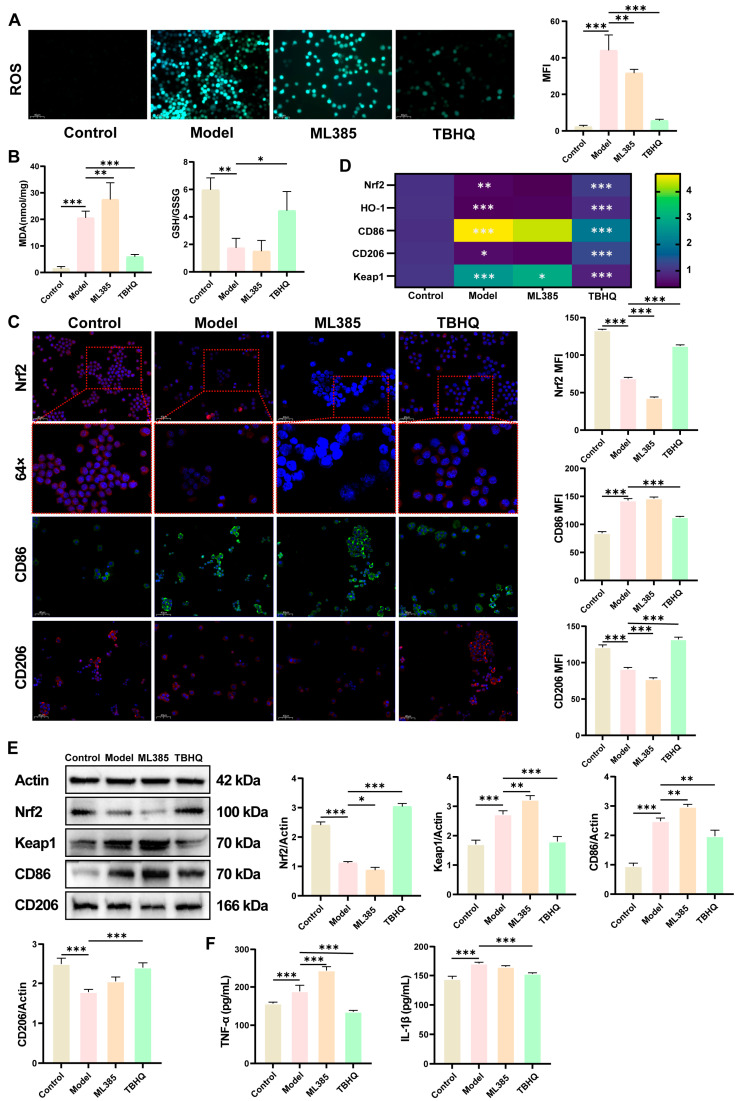
Nrf2 mediates the restoration of redox homeostasis and the reversal of macrophage polarization: (**A**) Representative fluorescence microscopy images showing intracellular ROS generation and accumulation in MH-S cells across all groups, accompanied by the quantitative analysis of mean fluorescence intensity (MFI). (**B**) Determination of intracellular MDA content (left panel) and the GSH/GSSG ratio (right panel) in each group using biochemical assay kits. Data in the bar graphs (**B**) are derived from six independent experiments (*n* = 6). (**C**) Immunofluorescence (IF) staining illustrating the expression and nuclear localization of Nrf2, alongside the intracellular expression abundance of M1 (CD86) and M2 (CD206) polarization markers; corresponding quantitative analyses of fluorescence intensity are shown on the right. (**D**) Gene expression heatmap illustrating the relative mRNA levels of antioxidant pathway components (Nrf2, HO-1, and Keap1) and polarization markers (CD86 and CD206) across all groups, as assessed by RT-qPCR. (**E**) Representative Western blot images and corresponding densitometric analyses displaying significant alterations in the protein levels of Nrf2, Keap1, CD86, and CD206. (**F**) Secretion levels of the pro-inflammatory cytokines TNF-α and IL-1β in cell culture supernatants, measured by ELISA. Data in the bar graphs (**A**,**C**–**E**) are presented as the mean ± standard deviation (SD) of three independent experiments. * *p* < 0.05, ** *p* < 0.01, *** *p* < 0.001.

Drawing upon established pharmacologically effective concentrations from the literature and our preliminary in vitro screenings, 10 µmol/L TBHQ and 5 µmol/L ML385 were selected as the optimal doses for subsequent intervention assays [25,26]. To evaluate the effects of TBHQ on SiO_2_-induced oxidative stress, intracellular levels of ROS, MDA, and the GSH/GSSG ratio were comprehensively quantified. The results demonstrated that TBHQ intervention effectively mitigated SiO_2_-induced ROS accumulation and MDA overproduction while successfully restoring the intracellular GSH/GSSG balance (Figure 2A,B). Subsequent evaluations focused on the expression dynamics of the Nrf2 signaling cascade. Compared to the SiO_2_-exposed model group, TBHQ significantly upregulated the mRNA expression of Nrf2 and its downstream antioxidant effector HO-1 while concomitantly downregulating Keap1 transcription (Figure 2D). Western blot analysis further corroborated these trends, confirming that TBHQ facilitated Nrf2 protein accumulation and effectively suppressed Keap1 protein levels (Figure 2E). Consistent with these findings, immunofluorescence imaging clearly visualized an enhanced intracellular Nrf2 fluorescence intensity following TBHQ treatment (Figure 2C). To elucidate the specific role of Nrf2 in macrophage polarization, the expression profiles of key phenotypic markers and pro-inflammatory cytokines were systematically evaluated. Compared to the Model group, TBHQ intervention effectively downregulated the M1 marker CD86 at both the transcriptional and translational levels, accompanied by a marked reduction in its fluorescence intensity (Figure 2C–E). Concurrently, TBHQ significantly curtailed the secretion of the pro-inflammatory factors TNF-α and IL-1β into the culture supernatant (Figure 2F). Notably, orthogonal assessments—encompassing gene quantification, protein analysis, and immunofluorescence imaging—consistently demonstrated that while SiO_2_ exposure suppressed the expression of the M2 marker CD206, TBHQ treatment successfully reversed this inhibition and restored robust CD206 expression (Figure 2C–E). Collectively, these findings indicate that Nrf2 activation effectively blunts SiO_2_-driven M1 polarization. Furthermore, the introduction of the specific Nrf2 inhibitor ML385 not only exacerbated intracellular oxidative stress (Figure 2A,B) but also completely abrogated the TBHQ-mediated suppression of the M1 marker (CD86) and pro-inflammatory cytokines, concomitantly blocking the restoration of CD206 (Figure 2C–F). This robust reverse validation solidifies the central role of the Nrf2 signaling axis as a pivotal therapeutic target for maintaining redox balance and driving anti-inflammatory homeostasis in alveolar macrophages.

### 3.3. Nrf2 Activation Reprograms the Glutamine Metabolic Profile of Macrophages Following SiO_2_ Stimulation

**Figure 3:**
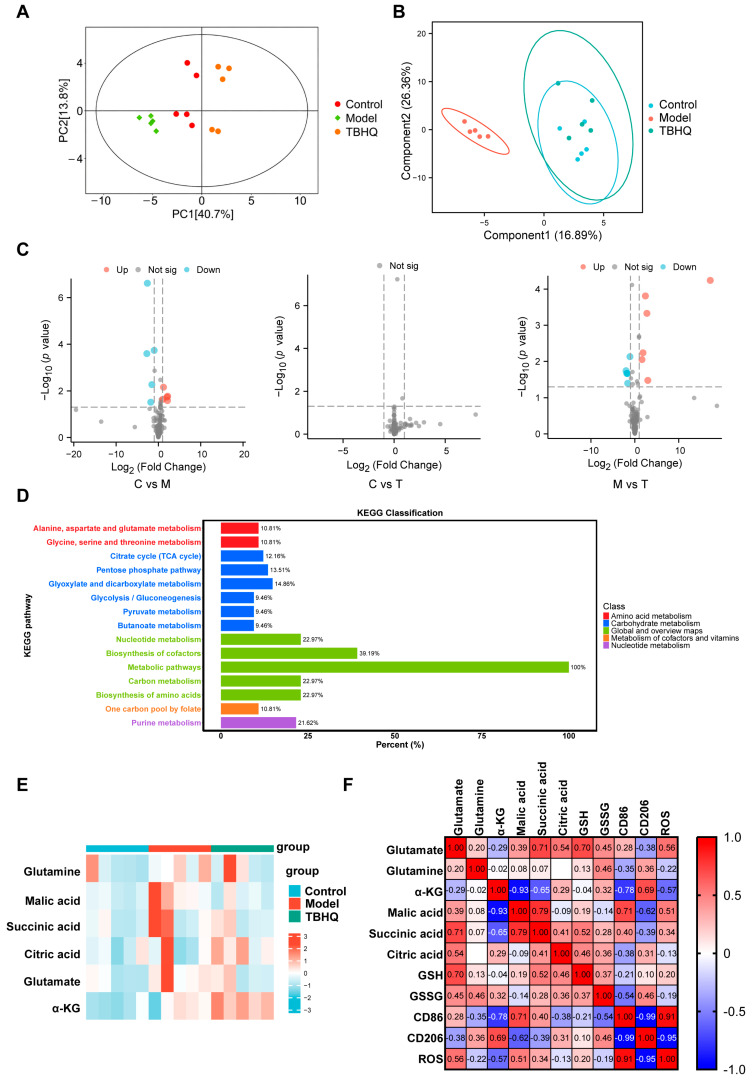
Targeted metabolomics reveals that Nrf2 activation reprograms glutamine metabolism in SiO_2_-stimulated macrophages: (**A**) Principal component analysis (PCA) score plot of cellular metabolites across all groups. (**B**) Partial least squares-discriminant analysis (PLS-DA) score plot, demonstrating significant spatial separation among the groups. (**C**) Volcano plots illustrating the distribution of differential metabolites in the control vs. model (C vs. M), control vs. TBHQ (C vs. T), and model vs. TBHQ (M vs. T) comparisons. (**D**) KEGG pathway enrichment plot of the differential metabolites. (**E**) Hierarchical clustering heatmap showing the relative abundances of core metabolites associated with glutamine metabolism and the TCA cycle. (**F**) Scatter plots depicting Spearman’s linear correlations among core indicators. The panels sequentially display: the positive correlation between the pro-inflammatory metabolite succinate and the M1 marker CD86, the positive correlation between the oxidative stress level (ROS) and glutamate, and the negative correlation between glutamine and the core product α-ketoglutarate (α-KG). Each group consists of five biological replicates (*n* = 5).

To systematically elucidate the SiO_2_-induced perturbations in glutamine metabolism within MH-S cells and the modulatory efficacy of TBHQ, a targeted metabolomics analysis was executed. Principal component analysis (PCA) and Partial Least Squares-Discriminant Analysis (PLS-DA) demonstrated a robust spatial separation between the model and control groups. Notably, the metabolic profile of the TBHQ-treated group showed a marked shift toward that of the control group (Figure 3A,B). A volcano plot further confirmed this re-shifting trend (Figure 3C). KEGG pathway enrichment analysis revealed significant enrichment in amino acid metabolism (particularly alanine, aspartate, and glutamate metabolism) and the TCA cycle (Figure 3D). In addition, we generated a clustering heatmap of key metabolites in the glutamine metabolic pathway. The results revealed increased expression of malate, succinate, citrate, glutamate, and the key node molecule α-ketoglutarate (α-KG) in the model group. TBHQ suppressed the abnormal accumulation of TCA cycle intermediates such as glutamate and citrate, bringing their levels back to near baseline, while α-KG remained at a relatively high abundance following the intervention (Figure 3E). To clarify the intricate interplay among oxidative stress, metabolic remodeling, and macrophage polarization phenotypes, a comprehensive correlation analysis was performed on key biomarkers (Figure 3F). The data demonstrated that intracellular ROS levels exhibited a robust positive correlation with both the M1 marker CD86 (r = 0.91) and glutamate (r = 0.56). Within the metabolic network, glutamate abundance was significantly positively correlated with the downstream TCA cycle intermediates succinate (r = 0.71) and citrate (r = 0.54). Furthermore, the TCA cycle intermediates malate (r = 0.71) and succinate (r = 0.40) displayed varying degrees of positive correlation with CD86 expression. These findings suggest that oxidative stress is tightly coupled with an aberrant accumulation of metabolites along the “glutamate–TCA cycle” axis and that the buildup of these specific intermediates is intimately linked to the M1 polarization phenotype. Collectively, these data underscore that Nrf2-mediated metabolic remodeling of the glutamine–TCA axis is directly implicated in the phenotypic regulation of alveolar macrophages under SiO_2_-induced stress.

### 3.4. Nrf2 Activation Regulates the Expression of Key Molecules Involved in Glutamine Metabolism in Alveolar Macrophages Following SiO_2_ Exposure

**Figure 4:**
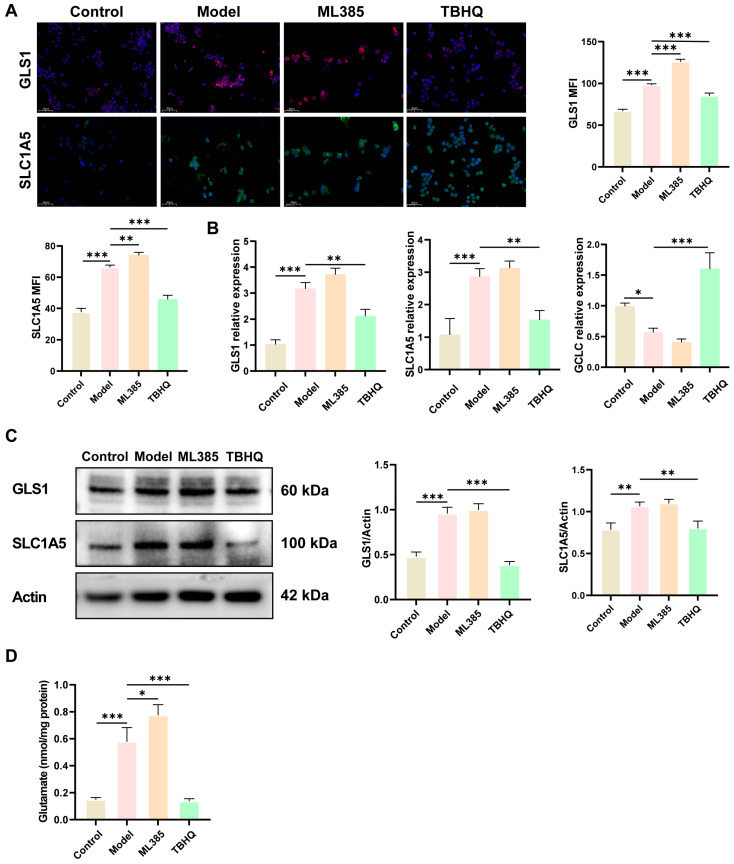
Nrf2 activation regulates key molecules involved in glutamine metabolism in SiO_2_-treated alveolar macrophages: (**A**) Representative immunofluorescence staining illustrating the expression and intracellular distribution of GLS1 and SLC1A5 across all groups, accompanied by the corresponding quantitative analysis of mean fluorescence intensity (MFI). (**B**) Relative mRNA expression levels of GLS1, SLC1A5, and GCLC in each group, assessed via real-time quantitative PCR (RT-qPCR). (**C**) Representative Western blot images and corresponding densitometric analyses demonstrating alterations in the protein levels of GLS1 and SLC1A5 across all groups. (**D**) Absolute intracellular glutamate (Glu) content in each group, quantified using a biochemical assay kit. Data are presented as the mean ± standard deviation (SD). Data in the bar graphs (**A**–**C**) are derived from three independent experiments, whereas data in (**D**) are from six independent experiments. * *p* < 0.05, ** *p* < 0.01, *** *p* < 0.001.

Building upon our targeted metabolomics findings, we sought to elucidate whether Nrf2 mechanistically regulates macrophage glutamine metabolism. To this end, we first evaluated the expression of the glutamine transporter SLC1A5 and the rate-limiting enzyme GLS1. Compared to the control group, SiO_2_ exposure significantly elevated the fluorescence intensities of SLC1A5 and GLS1, alongside a robust upregulation in their corresponding mRNA levels (SLC1A5 and GLS1). Simultaneously, SiO_2_ severely suppressed the mRNA expression of the downstream antioxidant enzyme GCLC. Notably, TBHQ treatment successfully reversed these phenotypic and transcriptional alterations (Figure 4A,B). Further mechanistic investigations revealed that TBHQ effectively downregulated the SiO_2_-induced protein expression of GLS1 and SLC1A5; however, when combined with the Nrf2 inhibitor ML385, the inhibitory effect of TBHQ was significantly reversed (Figure 4C). Subsequent quantification of the key intracellular metabolite Glu demonstrated that SiO_2_ exposure triggered a significant accumulation of Glu, whereas TBHQ intervention drastically reduced these intracellular levels (Figure 4D). Collectively, these data indicate that beyond alleviating SiO_2_-induced cellular damage, Nrf2 activation directly governs the expression of critical molecules dictating glutamine metabolism. These findings strongly suggest that Nrf2 reprograms SiO_2_-induced glutamine metabolic dysregulation fundamentally by inhibiting the SLC1A5/GLS1 axis and promoting GCLC synthesis.

### 3.5. The Protective Effect of Nrf2 Activation Against SiO_2_-Induced Alveolar Macrophage Damage Is Glutamine-Dependent

**Figure 5:**
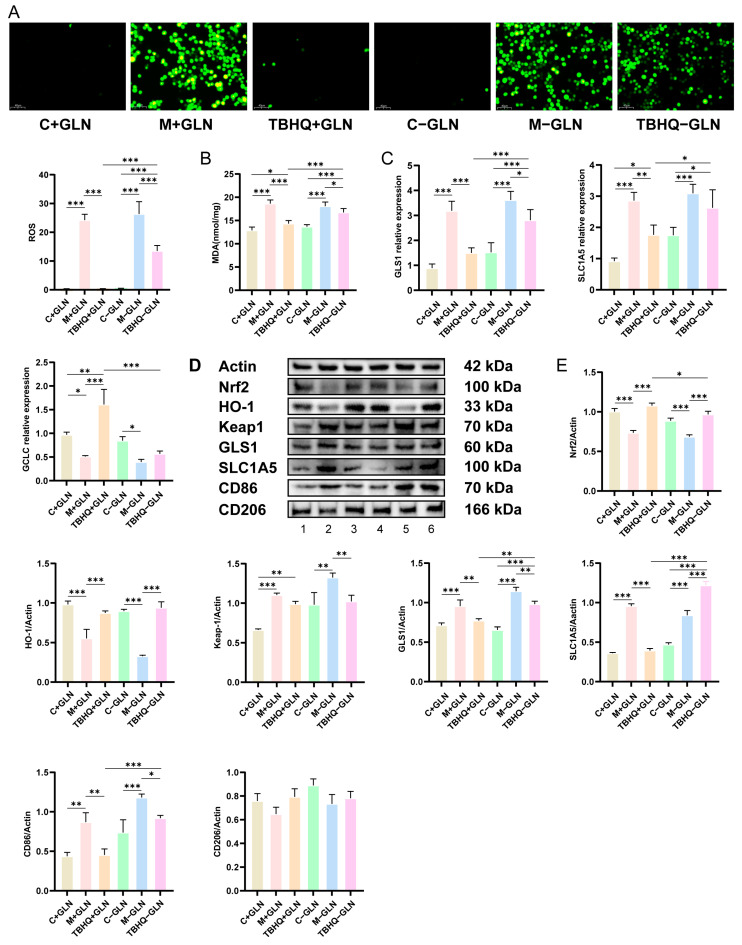
Effects of exogenous glutamine on TBHQ-mediated cytoprotection and polarization regulation in macrophages: (**A**) Representative fluorescence microscopy images showing intracellular ROS accumulation in MH-S cells across all groups under glutamine-replete (+Gln) or glutamine-deprived (−Gln) conditions, along with the corresponding quantitative analysis of mean fluorescence intensity (MFI). (**B**) Intracellular content of the lipid peroxidation marker MDA in each group, determined via a biochemical assay kit. Data in the bar graph (**B**) are derived from six independent experiments (*n* = 6). (**C**) Relative mRNA expression levels of glutamine metabolism-related genes (GLS1 and SLC1A5) and GCLC across all groups, assessed by RT-qPCR. (**D**) Representative Western blot images illustrating the protein expression of the intracellular Nrf2 signaling axis (Nrf2, HO-1, and Keap1), metabolic enzymes/transporters (GLS1 and SLC1A5), and polarization markers (CD86 and CD206). Lanes 1 to 6 sequentially represent the following groups: C + Gln, M + Gln, TBHQ + Gln, C − Gln, M − Gln, and TBHQ − Gln. (**E**) Densitometric quantification and statistical analysis of all target proteins corresponding to (**D**). Note: The TBHQ + Gln and TBHQ − Gln groups both represent TBHQ drug intervention groups established on the basis of the SiO_2_ model stimulation. Data in the bar graphs (**A**,**C**,**E**) are presented as the mean ± standard deviation (SD) of three independent experiments. * *p* < 0.05, ** *p* < 0.01, *** *p* < 0.001.

We evaluated the efficacy of TBHQ treatment under both Gln-replete (+Gln) and Gln-deprived (−Gln) conditions to determine the absolute necessity of exogenous Gln in Nrf2-mediated cytoprotection. The data revealed that TBHQ robustly promoted the protein expression of Nrf2 and HO-1 while downregulating Keap1, regardless of Gln availability in the culture medium (Figure 5D,E). This suggests that the early activation of the Nrf2 signaling pathway by TBHQ does not depend on the supply of exogenous Gln. However, the downstream protective effects orchestrated by Nrf2 activation were significantly compromised in the absence of Gln. Under Gln-deprived conditions, the capacity of TBHQ to scavenge SiO_2_-induced ROS and suppress MDA accumulation was markedly attenuated (Figure 5A,B). Notably, despite the sustained upregulation of upstream Nrf2 protein, the TBHQ-induced transcriptional enhancement of the key antioxidant enzyme GCLC was significantly blunted under −Gln conditions (Figure 5C). Regarding the molecular regulatory profile, TBHQ effectively suppressed the SiO_2_-driven upregulation of GLS1 and SLC1A5 under +Gln conditions; however, this inhibitory effect was drastically diminished upon Gln deprivation. Concomitantly, the suppressive effect of TBHQ on the M1 polarization marker CD86 was also weakened in the −Gln group (Figure 5D,E). Furthermore, within this specific experimental paradigm, no significant alterations in the protein expression of the M2 marker CD206 were detected across the groups. Collectively, these findings suggest that although TBHQ can independently activate Nrf2-upstream signaling, its ability to fully exert its protective functions—such as antioxidant effects (e.g., upregulation of GCLC) and inhibition of M1 polarization—depends to some extent on the presence of exogenous Gln.

## 4. Discussion

The massive generation of reactive oxygen species (ROS) by alveolar macrophages upon the phagocytosis of SiO_2_ particles represents a pivotal catalyst for the inflammatory cascade in early-stage silicosis [27,28]. During this process, inhaled SiO_2_ particles are typically phagocytosed and sequestered within lysosomes. As the crystalline surfaces possess highly reactive silanol groups, their direct contact with the lysosomal membrane rapidly destabilizes its structure, precipitating lysosomal rupture. This structural breach not only triggers the cytosolic leakage of lysosomal contents, such as cathepsins, but also coincides with a robust burst of intracellular ROS. Together, this intracellular damage and the marked accumulation of ROS serve as a pivotal early trigger for activating downstream pro-inflammatory cascades [6,29]. Importantly, inhaled SiO_2_ particles directly stimulate alveolar macrophages, the lung’s first line of innate defense, which serves as the primary trigger for their activation. While the complex in vivo microenvironment—notably through epithelial-derived DAMPs and cytokines—compounds this response [30,31], the direct SiO_2_–macrophage engagement remains the fundamental initiating event of this pathological network and the primary focus of this study. Under physiological conditions, the Nrf2/HO-1 signaling axis orchestrates robust antioxidant defenses to maintain cellular homeostasis; nevertheless, it is easily suppressed by acute dust exposure [15,32]. Our findings confirm that SiO_2_ exposure markedly suppresses Nrf2 expression and functional activity in macrophages. This inhibition precipitates severe oxidative stress, as evidenced by exacerbated lipid peroxidation and the depletion of endogenous reducing substrates, which is consistent with the findings of Wang, Yuan, et al. [33,34]. Consequently, this disruption of redox homeostasis drives a pro-inflammatory shift within the macrophages [8,35]. Collectively, the SiO_2_-induced compromise of the Nrf2 axis and the ensuing oxidative burden not only directly mediate cytotoxicity but also establish a critical prerequisite for the subsequent metabolic reprogramming of these cells.

The targeted activation of Nrf2 serves as a pivotal mechanism for restoring cellular redox homeostasis. Our results demonstrate that TBHQ treatment effectively drives Nrf2 activation and the transcription of its downstream target genes, thereby attenuating intracellular ROS overload and partially restoring cellular antioxidant capacity, which is consistent with the findings of Liu, Ye et al. [36,37]. Furthermore, given that aberrant ROS accumulation serves as a critical second messenger in propagating downstream inflammatory cascades [28,38,39], the antioxidant efficacy of TBHQ likely plays a fundamental role in intercepting early inflammatory signal transduction. Concomitant with the alleviation of the intracellular stress microenvironment, the SiO_2_-induced imbalance in macrophage polarization was markedly restored, characterized by the attenuation of pro-inflammatory responses and a partial transition toward a pro-repair phenotype [14,40]. To mechanistically validate this, we employed the specific Nrf2 inhibitor ML385. This loss-of-function approach confirmed that the beneficial modulation of the macrophage inflammatory phenotype is dependent on an intact Nrf2 signaling pathway. These results corroborate previous findings demonstrating that Nrf2 impairment blunts the protective efficacy of such interventions [15,32]. In summary, the reactivation of Nrf2 not only rectifies the cellular redox imbalance but also mitigates SiO_2_-provoked early damage by reprogramming macrophage polarization, thereby establishing a robust mechanistic rationale for the early prevention of related occupational lung diseases.

To comprehensively delineate the metabolic alterations underlying macrophage phenotypic shifts, targeted metabolomic profiling was performed. Our data revealed that SiO_2_ exposure triggered a marked intracellular accumulation of glutamate, α-ketoglutarate (α-KG), and key tricarboxylic acid (TCA) cycle intermediates, including succinate and citrate. These findings align with previous reports, further indicating that severe oxidative stress precipitates the characteristic “TCA cycle disruption” traditionally observed in activated macrophages [41,42,43]. Correlation analyses further elucidated the interconnectedness of oxidative stress, metabolic reprogramming, and macrophage polarization. Notably, the robust positive correlation between ROS and glutamate indicates that intracellular oxidative stress likely functions as an upstream driver of aberrant metabolite accumulation. Concurrently, TCA cycle intermediates—most prominently succinate—exhibited a strong positive association with the M1 phenotype marker CD86. These observations align seamlessly with current paradigms in immunometabolism, which posit that accumulated metabolites are not merely passive biochemical byproducts but actively operate as signaling molecules to orchestrate macrophage polarization [41,44,45]. For instance, the intracellular accumulation of succinate competitively inhibits prolyl hydroxylases (PHDs), leading to the stabilization of HIF-1α and the subsequent transcriptional induction of pro-inflammatory genes [44]. Conversely, upon its translocation to the cytosol, accumulated citrate fuels the biosynthesis of pro-inflammatory lipids and supplies essential substrates for NADPH oxidase, further amplifying the inflammatory cascade [46]. Our data demonstrate that Nrf2 activation via TBHQ partially ameliorates these metabolic perturbations, as evidenced by a marked decline in the accumulation of succinate, citrate, and glutamate. Although α-KG levels remained elevated post-intervention—likely reflecting a cellular compensatory mechanism to preserve the redox pool [41,45,47]—the predominant pro-inflammatory metabolites were effectively curtailed. While the present study lacks direct functional validation of individual metabolites via targeted genetic ablation, the robust metabolism–phenotype correlations observed herein strongly suggest a clear mechanistic trajectory. Specifically, targeting Nrf2 appears to relieve metabolic bottlenecks within the glutamine–TCA axis, thereby restricting the generation of pro-inflammatory signaling metabolites and, consequently, impeding the SiO_2_-induced pro-inflammatory polarization of macrophages.

Targeted metabolomic profiling revealed that SiO_2_ exposure precipitates the aberrant accumulation of key metabolites, notably glutamate, within alveolar macrophages. To elucidate the molecular mechanisms orchestrating this metabolic perturbation, we systematically evaluated the upstream transporter SLC1A5 and the rate-limiting enzyme GLS1 within the glutamine metabolic cascade. Under pro-inflammatory stress conditions, macrophages heavily depend on the SLC1A5/GLS1 axis to amplify glutamine cellular uptake and subsequent glutaminolysis, thereby supplying the requisite metabolic fueling for M1 polarization [48,49,50]. Molecular analyses in the present study definitively corroborated that SiO_2_ aberrantly hyperactivates the SLC1A5/GLS1 axis in alveolar macrophages, providing a mechanistic rationale for the profound glutamate accumulation observed in our metabolomic profiling. Interestingly, pharmacological activation of Nrf2 via TBHQ not only upregulated the antioxidant-associated enzyme GCLC but also profoundly blunted the overexpression of the SLC1A5/GLS1 axis. We hypothesize that this suppression stems primarily from indirect negative feedback driven by altered metabolic flux, rather than direct transcriptional repression. Specifically, the TCA cycle impairment induced by SiO_2_ exacerbates the depletion of intracellular metabolic pools, compelling macrophages to compensatorily upregulate SLC1A5 and GLS1 to sustain aberrant glutamine anaplerosis. Following TBHQ-mediated Nrf2 activation, this glutamine metabolic flux is effectively redirected from pro-inflammatory catabolism toward antioxidant anabolism [16,22,23,51]. Under these redirected conditions, cells preferentially channel substrates toward the biosynthesis of antioxidants, notably GSH. Concurrently, the restoration of intracellular redox homeostasis alleviates the exaggerated demand for TCA cycle anaplerosis. As the diversion of amino acids into pro-inflammatory pathways is halted, the overall cellular metabolic burden subsides. We propose that this physiological negative feedback loop ultimately drives the downregulation of SLC1A5 and GLS1. Nevertheless, whether Nrf2 also exerts direct transcriptional repression over these genes remains to be fully elucidated, warranting future validation through chromatin immunoprecipitation (ChIP) assays. Importantly, the introduction of the Nrf2 inhibitor ML385 profoundly abrogated the intervention’s modulatory effects on these key metabolites. This loss-of-function evidence demonstrates that the protective role of Nrf2 extends well beyond canonical free radical scavenging. Rather, it rectifies the macrophage polarization imbalance at a foundational metabolic level by actively reprogramming glutamine flux.

In the paradigm of macrophage polarization, elucidating the intricate crosstalk between intracellular signaling cascades and metabolic flux is of paramount importance. In our specific glutamine deprivation assays, we observed that even in the absence of exogenous glutamine, TBHQ robustly upregulated the protein expression of Nrf2 and HO-1, as anticipated. However, its capacity to scavenge intracellular ROS and suppress M1-driven pro-inflammatory polarization was significantly compromised. These findings are consistent with previous studies [52,53]. At the same time, the transcription of GCLC, a key antioxidant enzyme, was found to be dependent on substrate availability. This pronounced uncoupling between upstream signaling activation and downstream functional execution suggests that the mobilization of the Nrf2 transcription factor serves merely as the initial trigger for cellular defense responses. To fully actualize comprehensive antioxidant protection and reprogram the pro-inflammatory state of macrophages, an adequate supply of amino acid substrates remains indispensable [43,54]. This phenomenon partly elucidates why, under conditions of glutamine restriction, upstream signal activation alone remains insufficient to effectively abrogate SiO_2_-induced sustained cellular injury. Importantly, this loss of protective efficacy likely does not merely reflect a generalized energetic collapse but rather highlights a distinct dependency on the glutamine metabolic axis. During SiO_2_-induced pro-inflammatory activation, macrophages undergo significant metabolic rewiring, including TCA cycle disruptions, which substantially limit the capacity of glucose-derived carbons to efficiently convert into glutamate [41]. Because Nrf2-mediated glutathione (GSH) biosynthesis heavily relies on glutamate as a core precursor, these metabolically shifted cells become highly dependent on SLC1A5/GLS1-driven glutamine anaplerosis to replenish and maintain their antioxidant substrate pools [52]. Integrating these observations with our preceding data, the present study reveals that the cellular pro-inflammatory cascade is fundamentally reliant on glutamine utilization. Consequently, this strongly implies that Nrf2-targeted therapeutic interventions may derive their primary cytoprotective efficacy by systematically restricting the metabolic output that fuels this pro-inflammatory pathway.

It is important to acknowledge certain limitations in the present study. First, our substrate deprivation models relied primarily on in vitro manipulations of the culture medium composition, which may not fully recapitulate the highly intricate metabolic networks present in vivo. In future investigations, the incorporation of stable isotope tracing technologies (e.g., ^13^C-glutamine) will be imperative to quantitatively map the dynamic metabolic fluxes within macrophages. Furthermore, the deployment of specific gene-knockout animal models will be crucial to comprehensively validate the pathophysiological and translational significance of this intertwined signaling and metabolic axis in early in vivo interventions following silica dust exposure. However, the clinical translation of this early intervention strategy remains hindered by substantial barriers. First, systemic Nrf2 activation lacks target specificity, risking severe off-target effects such as shielding aberrant cells from apoptosis. Second, conventional systemic administration struggles to achieve therapeutic accumulation within alveolar macrophages amidst the complex pulmonary microenvironment. Consequently, alongside deeper mechanistic investigations, future development of localized delivery platforms—such as inhalable liposomes or macrophage-targeted nanocarriers—is imperative to maximize efficacy and circumvent systemic adverse effects.

## 5. Conclusions

In summary, the present study demonstrates that Nrf2 activation effectively mitigates SiO_2_-induced oxidative damage and M1-type pro-inflammatory polarization in alveolar macrophages. Crucially, this cytoprotective efficacy arises not merely from the direct scavenging of reactive oxygen species by Nrf2 but is fundamentally driven by its profound regulation of cellular glutamine metabolism. Mechanistically, Nrf2 orchestrates the suppression of the upstream transporter SLC1A5 and the rate-limiting enzyme GLS1, thereby effectively preventing the aberrant accumulation of intermediate metabolites such as glutamate and succinate. This regulatory intervention essentially re-channels the glutamine metabolic flux away from fueling pro-inflammatory cascades and toward the biosynthesis of vital antioxidants (e.g., GSH). Moreover, our substrate deprivation assays corroborated that the comprehensive antioxidant and anti-inflammatory functions of Nrf2 are intrinsically dependent on the availability of exogenous glutamine. Collectively, this study elucidates the pivotal role of Nrf2 in bridging the oxidative stress–metabolism–polarization axis. These findings strongly suggest that therapeutically targeting Nrf2 and its coupled glutamine metabolic network may yield promising novel strategies for the clinical prevention and early intervention of occupational respiratory diseases, particularly silicosis.

## Figures and Tables

**Table 1:** Quantitative real-time PCR primer sequences.

Gene	Primer Sequences (5′ to 3′)
Nrf2	Forward: CAGAGTGATGGTTGCCCACT
	Reverse: CACACACTTTCTGCGTGCTC
HO-1	Forward: AGACCGCCTTCCTGCTCAAC
	Reverse: GACGAAGTGACGCCATCTGTG
Keap1	Forward: TGGTCGCCCTGTGCCTCTATG
	Reverse: ATGCCACTCGTCCCGCTCTG
CD86	Forward: ATCTGCCGTGCCCATTTACA
	Reverse: CAACTTTTGCTGGTCCTGCC
CD206	Forward: AAATGGCTTCCTGGAGAGCC
	Reverse: ACCCTCCGGTACTACAGCAT
GLS1	Forward: CCAGTTCGCCCTCGGAGATC
	Reverse: CTGCTGCTGCTGCTGCTG
SLC1A5	Forward: GCCATCACCTCCATCAACGACTC
	Reverse: GGAAGGCAGCAGACACCAGATTG
GCLC	Forward: AGCTCCTGGAGGAAGGCATCG
	Reverse: GGTCAGACTCGTTGGCATCATCC
β-actin	Forward: ACTGCCGCATCCTCTTCCTC
	Reverse: AACCGCTCGTTGCCAATAGTG
GAPDH	Forward: GCAAATTCAACGGCACAGTCAAG
	Reverse: TCGCTCCTGGAAGATGGTGATG

## Data Availability

The original contributions presented in this study are included in the article. Data are available upon request from the corresponding author.
